# A decision support system for institutional support to farmers in the face of climate change challenges in Limpopo province

**DOI:** 10.1016/j.heliyon.2020.e04989

**Published:** 2020-11-03

**Authors:** Priscilla Ntuchu Kephe, Kingsley Kwabena Ayisi, Brilliant Mareme Petja

**Affiliations:** a Department of Geography and Environmental Studies, University of Limpopo, Private Bag X1106, Sovenga, 0727, Polokwane, South Africa; b Risk and Vulnerability Science Centre, University of Limpopo, Private Bag X1106, Sovenga, 0727, South Africa; c Water Research Commission, Private Bag X03, Gezina, 0031, Pretoria, South Africa

**Keywords:** Geography, Environmental sciences, Agricultural sciences, Institutional support, Adaptive capacity, Support institution, Cooperative governance, Climate change

## Abstract

Smallholder farmers in South Africa continue to be affected by the changing climate despite the existence of support to improve their adaptive capacity. This study focused on the institutional support systems and support types available to farmers in agro-ecological zones of Limpopo Province and assessed support types best suited to each area. Six hundred farmers were purposively sampled across the agro-ecological zones of Limpopo and interviewed. Support types looked at included monetary, machinery, seeds, educational support and others (irrigation scheme, animals, fertilizer, pesticides). Supporting institutions included Agro finance institutions, DAFF, Banks, and NGOs. Results showed that 70.01% of farmers received support from DAFF 25.60% from NGO's and 4.39% from Agro finance institutions. The most number of support received was two types 33.3% of the farmers. The result from the ANOVA showed that there were no significant differences in the level of difficulty experienced by farmers in accessing the various support institutions across the agro-ecological zones. In terms of the various support types received, there was a statistically significant difference in seeds (p = 0.002 < α = 0.05) and educational (p = 0.0001 < α = 0.05) support received between the different areas. Furthermore, the support needs varied across zones with farmers in arid-zone needing machinery, education, seeds and lastly monetary support while the semi-arid zone needed machinery, education, others, seeds, monetary and the humid, machinery, education, others, money and seeds. It is therefore recommended that support for farmers should be location-specific in order to enhance the adaptive capacity of an area and not be based only on the availability of certain support. There is a need for proper coordination between institutions in their aim to assist farmers to cope with climate change.

## 1 Introduction

Institutional support across the Limpopo province needs to be structured in such a way that farmers get the much-needed assistance to continue producing under a changing climate. According to Maponya and Mpandeli (2012), institutional support, both technical and financial (e.g. inputs, technology transfer and capacity building, drought mitigation, historical rainfall distribution information and market trends) are important factors which influence farmers' productivity. The majority of farmers in the Limpopo province are resource-poor (Maponya and Mpandeli (2012)) who are highly dependent on the services of Agricultural Extension Officers. However, they do not have the required amount of inputs and resources necessary for optimal production. This limitation, coupled with the local effects from climate change (e.g. droughts of 1982/83, 1987/88, 1991/92, 1994/95, 2002/03, 2008/09 and 2015/16, floods: 1923, 1940, 1955, 1967, 1980, 2000, 2011, 2013, 2014, 2016 (Agricultural Disaster Management Policy, 2011; Bureau for Food and Agricultural Policy (BFAP), 2011) as well as bushfires increase the vulnerability of the farmers. Besides climate change other challenges facing farmers in the Limpopo Province have been well documented (e.g. Azan and Besley, 1991; Makhura, 2001). These challenges place the farmers in a difficult situation, where they are unable to produce enough to generate income to undertake various adaptation options. The challenges are not unique to Limpopo province alone, as they have been cited to affect other provinces such as the Eastern Cape, Mpumalanga, Free State as well (Mpandeli, 2006).

Addressing the challenges faced by farmers is paramount if sustainable productivity is to be carried under the current climate stresses (Cherotich et al., 2012) and projected climate change scenarios. Adoption of appropriate interventions and support services will provide an opportunity for the farmers to withstand the climatic challenges hence strengthening their capacity towards effective production. Studies such as those of Deressa et al. (2009), Kabubo-Mariara (2008); Mideksa (2009); Bryan et al. (2009) have shown that several environmental, socio-economic, institutional factors as well as the economic structure are key drivers which influences the farmers to choose specific adaptation methods in Africa and especially Sub Saharan African countries.

Adaptation programs have been developed around the world to create institutions and infrastructure for guiding responses to climate change. According to Eriksen et al. (2015) the creation of such institutions as adaptation measures, brings about the formation of rational policy interventions as well as planned responses to actual or expected biophysical changes as a result of climate change. This framing of adaptation is restrictive in that in conceptualising adaptation as a policy process the people become the ‘recipients of adaptation’, instead of being active agents of adaptation (Eriksen et al., 2015). Furthermore framing adaptation as a response to direct biophysical climatic impacts causes the neglect of other important but indirect effects that occur from social and physical interactions (O'Brien et al., 2006). According Biagini et al. (2014) and Eakin and Patt (2011) once vulnerabilities are identified, there is a shift in focus geared towards technical measures such as infrastructure and institutional design including new coordination bodies from nation to regional levels as well as community-based environmental management groups Adaptation programmes are therefore fundamentally underpinned by the assumption that that the vulnerability to climate change is as a resut of the combination of biophysical change and marginalization. Hence the best way to adapt is through a variety of institution-building technical and measures.

Adaptation projects therefore attempt to bring stakeholders operating at different levels into cooperative arrangements (institutions) inorder to administer resources that cut across current jurisdictional boundaries (Agrawal and Perrin, 2009; Bulkeley, 2016; Eakin and Lemos, 2010; Stripple and Bulkeley, 2013). While the promotion of cooperative arrangements sounds perfectly reasonable, in many contexts, however according to Nightingale (2017) it is these institutional rules and relationships precisely that are intensely contested. Forsyth (2005) states that eventhough design principles of instituions are important, the success or failure of the institutions has less to do with them. Rather the success or failure of institution is dependent on the interplay social-political struggles within them (Forsyth, 2005). This shows that institutions are infused with power and politics with potentially a very large terrain of governance (see Eriksen et al., 2015). Climate change adaptation measures attempts at managing changing resources which can instead lead to an increase in conflict over the governance of these resources rather than rationalizing their use (Eriksen et al., 2015). This therefore means that programs aimed at alleviating vulnerability can instead exacerbate it. In internationally sponsored climate change adaptation contexts, however, the focus is on: (i) formal government and governance institutions intended to guide and foster positive change (Adger et al., 2009), and (ii) the relationships between these institutions and people targeted for adaptation efforts.

Adger et al. (2009) are of the opinion that often, adaptation to climate change is limited by the values, perceptions, processes and power structures within society. What may be a limit in one society may not be in another, depending on the ethical standpoint, the emphasis placed on scientific projections, the risk perceptions of the society, and the extent to which places and cultures are valued. In the case where power and politics overshadows and reshapes the whole purpose of adaptation efforts, then adaptation efforts becomes as aptly put by Nightingale (2017) more about adjusting to entangled socio-political contestations, biophysical change, livelihood desires, struggles for authority to govern change, and desires for social and political recognition by both those promoting programs and recipients of them. This goes to undermine the whole adaptation efforts from institutions to the vulnerable.

Climate adaptation programs in South Africa and Limpopo, have been designed with an understanding of rural livelihoods in mind and as such do not address the various dimensions of vulnerability and how they shape adaptation for different farmers practicing rainfed agriculture. It is therefore highly problematic that adaptation programs are not designed to specifically query how social, economic and political settings will affect adaptation and what kind of support areas should be having. There is, therefore, a need for each key stakeholder to understand the scope and drivers of measures of adaptation to climate change particularly amongst the smallholder farmers within their jurisdiction. This will enable them to develop and put in place the appropriate infrastructure and institutional support structures such as wider smallholder support programmes. Such support types include but are not limited to land access, extension and training, production support, input supply, mechanisation, irrigation, financing, infrastructure, and market support. A thorough understanding of the scope and drivers of measures of adaptation to climate change by key stakeholders particularly amongst the smallholder farmers within their jurisdiction is thus a critical approach to develop and put in place the appropriate measures and institutional support structures to minimize climate impacts. This is necessary, given that the vulnerability and sensitivity of each country or region to climate change differ and so does the accessibility of the different adaptation methods.

The South African government is aware of the stresses associated with agriculture especially rainfed agriculture and amongst smallholder and subsistence farmers. In its effort to combat the effects of climate change, poverty and enable sustainable food production especially amongst the smallholder farmers, the government has put in place support systems and programmes to assist farmers. Agricultural support from various levels of government has been shown to operate concurrently and sometimes share the responsibility between national and provincial governments as well as non-governmental institutions. This means therefore that the various institutions have programmes in agriculture gearing towards the support of farmers. For example, DAFF provides conditional grants for provinces to carry out national programmes. DAFF's Programme 3: Food Security and Agrarian Reform has as one of its medium-term objectives as the provision of production inputs, such as seed and fertiliser, to increase the number of households benefiting to 200,000 by March 2021; and ii) cultivate 360,000 ha of underutilised land in communal areas and land reform projects for food (National Treasury, 2018). Several programmes such as the Comprehensive Agricultural Support Programme (CASP), Revitalisation of small-scale Irrigation Schemes (RESIS) all have the intention to assist farmers through one type of support or another. Besides, the department sees extension services as '*an amorphous umbrella term to describe all activities that combine information and advisory services needed and demanded by farmers*' (DAFF, 2011). The effectiveness of extension services in supporting smallholder farmers should be seen in the mobilisation of the social capital of communities (Rivera et al., 2001; Ferris et al., 2014). The implication thereof is the need to bring together farmers with similar circumstances (e.g. factors causing vulnerability, the scale of production), manage them and ensure they benefit from this synergy as well as making it easier for training and sharing of information.

On their part, the Department of Rural Development and Land Reform (DRDLR) has its farmer support programmes which it implements directly, working with provinces, other departments, and other stakeholders. Provinces also use their budgets, including the provincial equitable share (PES) from National Treasury to carry out their own programmes and to supplement funds for national programmes.

Furthermore, cooperatives in South Africa operate across different sectors of the economy, including the agricultural sector, the consumer's sector, financial services as well as worker cooperatives (Ncube, 2017). In the case of the agricultural sector, the agricultural cooperatives have assisted their members in accessing inputs (e.g. seed, fertilizer, pesticides, and credit) in bulk, have provided advisory services; tillage services, produce bulk up, transportation as well as linkages with the markets (Ortmann and King, 2007). The aim of establishing cooperatives is to serve the agricultural needs of farmers around areas of access to resources, output markets as well as providing a legal and operational framework (Ortmann and King, 2006; DAFF, 2012). All the support types offered have an intended benefit to be obtained. Thus, if farmers can have access to adequate support services, rainfed agriculture can make a difference by increasing agricultural growth, farm income and improved livelihood.

Studies have been conducted to investigate the negative impacts of climate change on smallholder farmers and their adaptation strategies (e.g. Hosu et al., 2016; Ubisi, 2016) and on institutional support in Limpopo (e.g Mapony and Mpandeli, 2012), however, there is very little information on the support type available to farmers in Limpopo and how accessible these supports are. Sufficient work has not been done with regards to assessing farmer support programmes in practice in South Africa in general and Limpopo in particular, even though R46 billion has been allocated to provincial farmer support programmes from 2010 to 2020 and stated objectives of government programmes set to provide support to 300,000 smallholder farmers. Furthermore, there is very little information on the institutions offering such supports. Therefore, this study aims to explore the types and number of supports available to smallholder farmers from various institutions aimed at bolstering their adaptation strategies towards climate change interventions and support systems in Limpopo province. It further aims to recommend suitable supports that can be geared where they make the most difference.

## 2 Materials and methods

### 2.1 Description of the study area

#### 2.1.1 Climate

This study was carried out in the Limpopo province of South Africa. The province is made up of four distinct climatic zones. These are the subtropical plateau characterised by a flat elevated interior area hot and dry with winter rain; a moderate eastern plateau with warm to hot and rainy summers and cold dry winters; the escarpment region with colder weather because of the altitude and rain all year round and the subtropical Lowveld region with hot-rainy summers and warm-dry winters.

Rainfall in the province occurs mostly between the months of October to April and ranges from 200mm in the hot dry areas to 1500mm in the high rainfall areas. There is high annual rainfall variability in the provinceas well as extreme weather events (Tennant and Hewitson, 2002; Cook et al., 2004). The three climatic regions in the provinve include (i) the Lowveld region characterised by a semi-arid climate(s), (ii) the Middle and Highveld classified as semi-arid, and (ii) Escarpment that experiences sub-humid climate (Limpopo Department of Agriculture (LDA), 2008). The province experiences long sunny days and dry weather conditions on most days. The uneven rainfall distribution and high-temperature regimes in the province results in high evaporative water demand and generally low crop-water-use efficiency (Mzezewa et al., 2010). .

### 2.2 Data collection

A quantitative research method with close-ended questions was employed in this study. The aim was to compare responses across the participants because all participants were asked identical questions in the same order to allow for a significant comparison of responses across participants and study sites (Crossman, 2017). Questionnaires were administered to individual farmers to provide information on the support systems available to them to cope with the climatic and non-climatic challenges.

#### 2.2.1 Sampling technique

**Figure 1:**
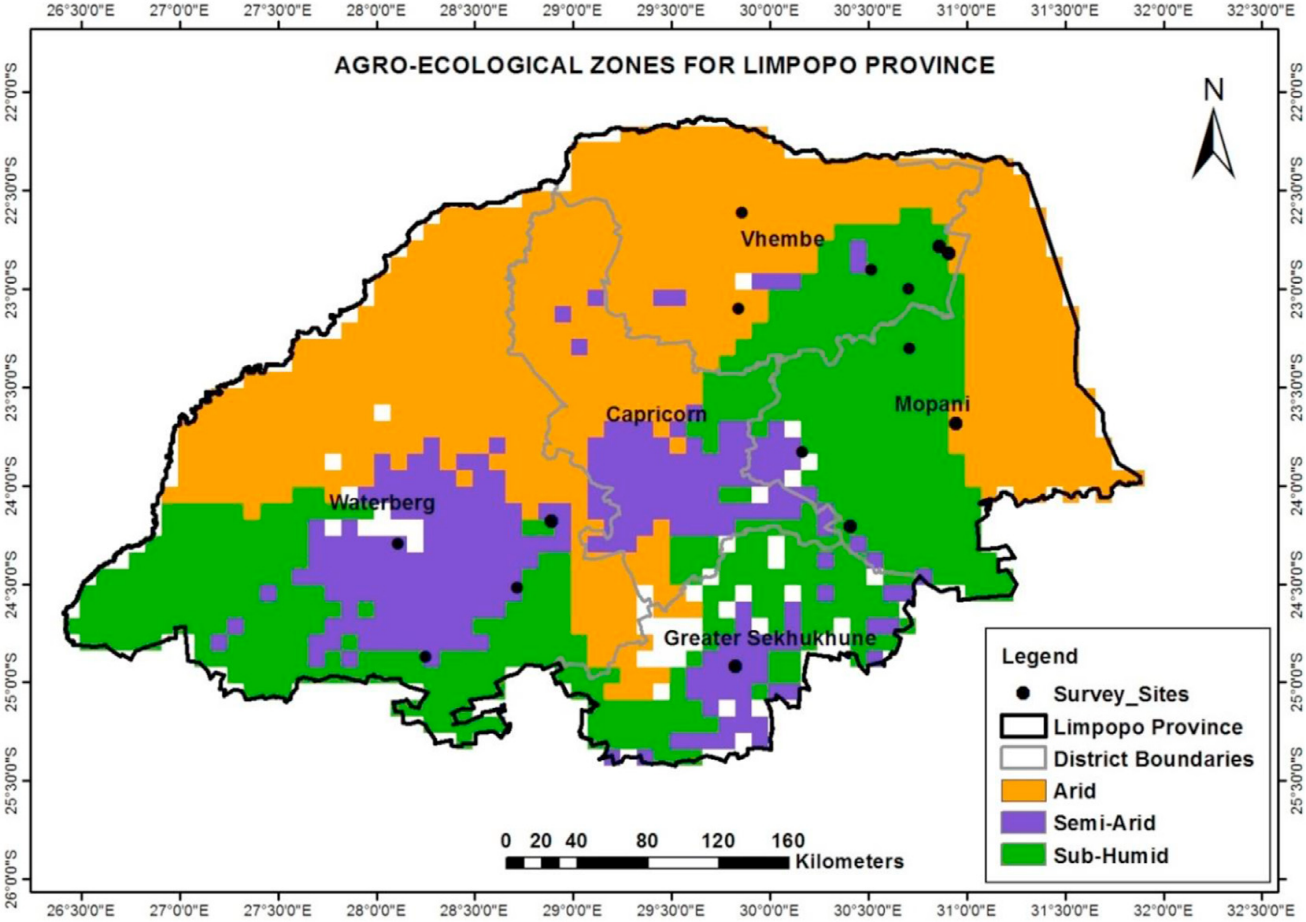
Agroecological zones for Limpopo Province and sample sites. Source: Adapted from Harvest Choice, 2010.

The population who participated in this study comprised of farmers residing in Limpopo province. A purposive random sample of 600 smallholder farmers participated in this study in order to cover different agro-ecological zones. 200 participants were targeted from each agro-ecological zone in the province (Figure 1). A criterion to select the participating sample was set as follows: the respondents were individual smallholder farmers, practicing crop production and depended on rainfall. Relevant departments in each local municipality provided a list fitting the stated criteria and smallholder farmers were randomly selected from bases on agro-ecological zones in Limpopo. The sampling was used to assess uniformity and homogeneous characteristics and to meet the objectives of the study, and it had to adhere to the statistical specifications for accuracy and representatively.

#### 2.2.2 Questionnaires

Questionnaires were used to collect information about climate change interventions and support systems in place in Limpopo and the ease with which they were made accessible to the farmers. The questionnaire was pre-tested in a pilot study so as to minimize errors and format unclear questions. A pilot study was conducted on a sample of farmers in each of the agro-ecological zones. This process helped in structuring the interview procedures and modifying the questions. In the pilot-testing process, space was provided for criticism and suggestions to improve the items. The questionnaire was tested on its usefulness, question clarity, language used, and consistency. After testing, the questionnaire was revised based on field experience and all corrections and advice incorporated. Farmers from the study population who were not included in the pilot study were interviewed to test the reliability of the instrument. All procedures performed in this study involving human participants were in accordance with the ethical standards of the University of Limpopo research committee and with the 1964 Helsinki declaration and its later amendments or comparable ethical standards. Informed consent was obtained from all individual participants included in the study.

Farmers were interviewed in their respective languages. An interview was the preferred data collection method for this study because it was anticipated that many farmers in the study area were unable to read and write. A hundred percent response rate was achieved given that interviews we scheduled to accommodate selected respondents in terms of availability and language of communication.

#### 2.2.3 Data analysis

Microsoft Excel 2010 statistical package and its add-on Xlstats 2018.2 were used to analyse the data. Collected data were coded to provide a general overview of which institutional support the farmers were receiving and how accessible they were. Descriptive statistics on the number of institutions giving support and the types of support received across the three agro-ecological zones in Limpopo was carried out. Statistical indicators such as frequencies, percentages, means, standard mean errors and standard deviation were computed. The focus on the descriptive statistics was on counts of farmers receiving supports, how many of the various support types were each farmer receiving and which institutions were doing the most in terms of farmer support as well as the variation in terms of support types and the number of support received per zone. A factor analysis was used to evaluate the support factors which were the most important. An analysis of variance (ANOVA) was done to show the variation between the types of support received per area. A test of significance was done to determine the similarity between zones.

## 3 Results

Results from this study aimed at answering the question on the types of support types available to farmers across the three agro-ecological zones in Limpopo, which institutions were offering such support and how support types and the total number of support varied across the zones as well the ease to which farmers accessed support types.

### 3.1 Support institutions and support types received by farmers

**Figure 2:**
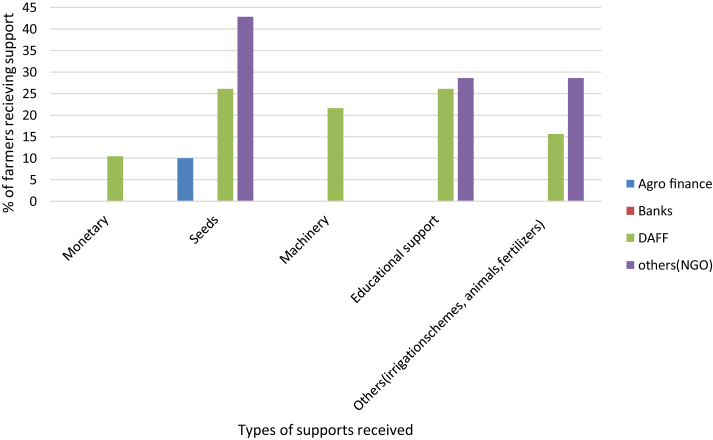
Types of support received and institutions giving such support type.

Table 1 and Figure 2 show the various types and sources of support received by the farmers in Limpopo. Support types the areas included monetary supports, seeds, machinery, educational support, and others. The institutions offering support to farmers in the area were Agro finance institutions, Banks, DAFF and NGOs. Results showed that DAFF gave 70.01% of support to farmers followed by NGOs (25.60%) and Agro finance institutions (4.39%). From the supports given by DAFF, 10.44% of the farmers received monetary support, 26.12% seeds, 21.63% machinery, 26.12% educational support and 15.67% from irrigation schemes, livestock and/or fertilizers. A total of 42.85 % of the farmers got seeds, 28.57% educational support and 28.57% got other types of supports from NGOs. It can be seen that most support received by farmers was in terms of seeds (66.67%) followed by educational and other types of support each at 41.67%.

**Table 1:** Support institutions and support types received by farmers across three agro-ecological zones in Limpopo.

Institution	Monetary		Seeds			Machinery			Education			Others			
	Arid	Semi-arid	Humid	Arid	Semi-arid	Humid	Arid	Semi-arid	Humid	Arid	Semi-arid	Humid	Arid	Semi-arid	Humid
Agro finance	0	0	0	12.5	7.5	10	0	0	0	0	0	0	0	0	0
Banks	0	0	0	0	0	0	0	0	0	0	0	0	0	0	0
DAFF	12.5	17.5	20	45	35	45	28.5	37.5	37.5	46.5	28.5	27.5	15	15	15
Ngo	0	0	0	27.5	22.5	25	0	0	0	20	20	10	16.5	15	19.5
**Total % of farmers receiving support**	**12.5**	**17.5**	**20**	**72.5**	**57.5**	**70**	**28.5**	**37.5**	**37.5**	**66.5**	**48.5**	**37.5**	**31.5**	**30**	**34.5**

Across the agro-ecological zones, results from Table 1 showed that in terms of monetary support across the zones, the arid area received the least support with only 12.5 of farmers receiving said support type. With regards to seeds, in the arid area, 72.5% received seeds making the area the area receiving most in terms of seed support, followed by the humid area with 70% of farmers. Areas receiving the most in terms of machinery support were the semi-arid and humid area. The highest education support was seen in the arid areas with DAFF supplying most of the support types. With the other types of support received, most of the farmers who received such supports were in the humid area (34.5%).

On average, the standard error of the mean was 0.040, which was quite low. The standard deviation was 0.575, which showed that there was low disparity among the respondents concerning the level of access to public agricultural extension and advisory services.

### 3.2 Number of supports received

The results in Table 2 indicate that amongst the total number of help types received, an average of 2.2% of farmers was not receiving any type of support. Amongst the three zones, the humid area had the highest percentage of farmers not receiving any form of support. The highest number of support types received was 2 types across the three zones with 33.3% and 25% receiving only one support type. Most farmers receiving all support types were found in the arid area (10.5%) followed by the semi-arid area (3.5%).

**Table 2:** Number of support types received by farmers across three agro ecological zones in Limpopo.

Number of Support type	% of farmers receiving support in Arid area	% of farmers receiving support in the semi-arid area	% of farmers receiving support in Humid area	% of farmers across all three zones receiving support
0	2	2	2.5	2.2
1	17.5	25.5	32	25.0
2	33	33	34	33.3
3	20	23	19	20.7
4	17	13	10	13.3
5	10.5	3.5	2.5	5.5
**Grand Total**	**100**	**100**	**100**	**100**

### 3.3 Ease of accessing support institutions

Results in Table 3 show that farmers experience challenges in accessing the support institutions. Only 5.8 % of the population can access the Agro Finance institution with ease with the humid area having most of the farmers accessing this institution with ease. All farmers in the area said it was very difficult to access banks, while 67% and 69.5% said it was difficult to access DAFF and NGOs respectively.

**Table 3:** Ease with which farmers access support institutions in three agro-ecological zone in Limpopo.

	% of farmers accessing support in Arid area	% of farmers accessing support in Semi-arid area	% of farmers accessing support in Humid	Total % of farmers accessing support
Agro finance	5	5	7.5	5.8
Banks	0	0	0	0
DAFF	34	31	34	33
NGO	29	29.5	33	30.5

### 3.4 Ease of accessing loans from a financial institution

Table 4 shows the result of the ease with which farmers can access loans from financial institutions in Limpopo. Results indicate it is very difficult for farmers to access such facilities. Across the three regions, above 60% of the farmers cannot access loans from financial institutions. In the arid areas 14% of the farmers can access loans, while in the semi-arid and humid, it showed 16% and 20% respectively.

**Table 4:** Ease with which farmers acess financial support from financial institutions in three agro-ecological zones in Limpopo.

Institutions	Very easy			Somewhat easy			Easy			Not very easy			Not easy at all		
	Arid area	Semi-arid area	Humid area	Arid area	Semi-arid area	Humid area	Arid area	Semi-arid area	Humid area	Arid area	Semi-arid area	Humid area	Arid area	Semi-arid area	Humid area
Agro finance	0	0	0	0	0	0	0	0	0	35	31.5	33.5	69	66.5	64.5
Banks	0	0	0	0	0	0	0	0	0	41	31.5	31.5	66	65	65
cooperatives	0	0	0	0	0	0	14	16	20	17.5	21	20	63.5	66.5	61.5

### 3.5 Variation in support types across institutions and agro-ecological zones

Tables 5 and 6 show the output of the ANOVA analysis and whether there is a statistically significant difference between the ease with which farmers across the various agro-ecological zones had access to support institutions and support types respectively. Results in Table 5 show a significant of 0.47 for Agro Finance, 0.55 for DAFF and 0.68. Given that these values of significance are greater than 0.05 (i.e., p = 0.05), therefore, there is a statistically insignificant difference in the level of difficulty in accessing the various support institutions across the agro-ecological zones.

**Table 5:** ANOVA on the ease with which farmers get support from different support institutions in three agro-ecological zones in Limpopo.

Variation in ease of accessing Agro Finance institutions						
SUMMARY						
Groups	Count	Sum	Average	Variance		
Arid Agro finance	200	10	0.05	0.047739		
Semi-Arid Agro finance	200	10	0.05	0.047739		
Humid Agro finance	200	15	0.075	0.069724		
**ANOVA**						
**Source of Variation**	**SS**	**df**	**MS**	**F**	**P-value**	**F crit**
Between Groups	0.083333333	2	0.041667	0.756654	0.469683	3.010815
Within Groups	32.875	597	0.055067			
Total	32.95833333	599				

**Variation in ease of accessing DAFF**						
SUMMARY						
Groups	Count	Sum	Average	Variance		
Arid DAFF	200	55	0.275	0.200377		
Semi arid DAFF	200	46	0.23	0.17799		
Humid DAFF	200	48	0.24	0.183317		
**ANOVA**						
**Source of Variation**	**SS**	**df**	**MS**	**F**	**P-value**	**F crit**
Between Groups	0.223333333	2	0.111667	0.596421	0.551107	3.010815
Within Groups	111.775	597	0.187228			
Total	111.9983333	599				

**Variation in ease of accessing NGO**						
ANOVA: Single Factor						
SUMMARY						
Groups	Count	Sum	Average	Variance		
Arid NGO	200	59	0.295	0.20902		
semi arid NGO	200	59	0.295	0.20902		
Humidv NGO	200	66	0.33	0.222211		
**ANOVA**						
**Source of Variation**	**SS**	**df**	**MS**	**F**	**P-value**	**F crit**
Between Groups	0.163333333	2	0.081667	0.382662	0.68221	3.010815
Within Groups	127.41	597	0.213417			
Total	127.5733333	599

**Table 6:** ANOVA on the variation in the number of support types received by farmers in three agro-ecological zones in Limpopo.

Variation in						
SUMMARY						
Groups	Count	Sum	Average	Variance		
Arid	200	25	0.125	0.109925		
Semi-arid	200	35	0.175	0.145101		
Humid	200	40	0.2	0.160804		
ANOVA						
Source of Variation	SS	df	MS	F	P-value	*F crit*
Between Groups	0.583333	2	0.291667	2.10423	0.122843	3.010815
Within Groups	82.75	597	0.13861			
Total	83.33333	599				

**Variation in seed support**						
SUMMARY						
Groups	Count	Sum	Average	Variance		
Arid	200	146	0.73	0.19809		
Semi-arid	200	115	0.575	0.245603		
Humid	200	140	0.7	0.211055		
ANOVA						
Source of Variation	SS	df	MS	F	P-value	*F crit*
Between Groups	2.703333	2	1.351667	6.193215	0.002177	3.010815
Within Groups	130.295	597	0.21825			
Total	132.9983	599				

**Variation in machinery support**						
SUMMARY						
Groups	Count	Sum	Average	Variance		
Arid	200	57	0.285	0.204799		
Semi-arid	200	75	0.375	0.235553		
Humid	200	75	0.375	0.235553		
ANOVA						
Source of Variation	SS	df	MS	F	P-value	*F crit*
Between Groups	1.08	2	0.54	2.396788	0.091885	3.010815
Within Groups	134.505	597	0.225302			
Total	135.585	599				

**Variation in educational support**						
SUMMARY						
Groups	Count	Sum	Average	Variance		
Arid	200	133	0.665	0.223894		
Semi-arid	200	98	0.49	0.251156		
Humid	200	76	0.38	0.236784		
ANOVA						
Source of Variation	SS	df	MS	F	P-value	*F crit*
Between Groups	8.263333	2	4.131667	17.41276	0.0001	3.010815
Within Groups	141.655	597	0.237278			
Total	149.9183	599				

**Variation in other support**						
SUMMARY						
Groups	Count	Sum	Average	Variance		
Arid	200	63	0.315	0.216859		
Semi-arid	200	60	0.3	0.211055		
Humid	200	69	0.345	0.227111		
ANOVA						
Source of Variation	SS	df	MS	F	P-value	*F crit*
Between Groups	0.21	2	0.105	0.480898	0.618468	3.010815
Within Groups	130.35	597	0.218342			
Total	130.56	599				

Table 6 shows that support types in terms of monetary (.012), machinery (0.09) and other support types (0.62) there was no statistically significant difference between these support types across the different agro-ecological zones given that the values were higher than *p* = .005. However, there are a statistically significant difference in seeds (0.002) and educational (0.0001) support types received between the different areas. This is so given that the significance values obtained are than p = 0.05.

### 3.6 Support types needed by farmers

The Kaiser-Meyer-Olkin (KMO) (Table 7) was used to assess sampling adequacy and evaluation of any correlations, which is acceptable at values >0.500. The result from Table 7 shows a KMO value of .0.67, 0.78 and 0.81 for the Arid, Semi-arid and humid areas respectively. This means the sample data could be used to perform a factor analysis. The Cronbach's alpha was 0.84(Arid), 0.90(Semi-arid) and 0.91(Humid) suggesting that the sample is statistically correlated with high reliability.

**Table 7:** Kaiser-Meyer-Olkin (KMO) measure of sampling adequacy of support types in three agro-ecological zones in Limpopo.

Kaiser-Meyer-Olkin measure of sampling adequacy:					
Arid area		Semi-arid area		Humid area	
**Monetary**	0.919764	**Monetary**	0.848594889	**Monetary**	0.933999
**Seeds**	0.619045	**Seeds**	0.772512764	**Seeds**	0.991345
**Machinery**	0.650632	**Machinery**	0.796388944	**Machinery**	0.724495
**Education**	0.641534	**Education**	0.755728782	**Education**	0.746629
**Others (irrigation, animals, fertilizers)**	0.66712	**Others (irrigation, animals, fertilizers)**	0.767608353	**Others (irrigation, animals, fertilizers)**	0.917998
**KMO**	0.671713	**KMO**	0.782722337	**KMO**	0.819925
**Cronbach's alpha:**	0.842453	**Cronbach's alpha:**	0.904264241	**Cronbach's alpha:**	0.908416

As shown in Table 2 it is not always going to be possible for all farmers to receive more than one type of support. But if institutions were to work together, they could make sure that each farmer received some type of support or better still more than one support type. Factor analysis was used to get the most important help type needed by farmers as well as factors that can work together to enhance farmer's adaptive capacity. Results from the factor analysis, Table 8, show that machinery support was the most important factor towards farmer's adaptive capacity across all three zones.

**Table 8:** Factor Analysis of support types in three agro-ecological zones in Limpopo.

Area	Support type	F1	F2	Initial communality	Final communality	Specific variance
Arid	Monetary	**0.539012**	0.260437	0.358396	0.358361	0.641638554
	Seeds	**0.70481**	-0.58056	0.734206	0.833805	0.166195147
	Machinery	**0.900081**	0.434636	0.876024	0.999054	0.000946069
	Education	**0.764503**	-0.5478	0.753925	0.884553	0.115446629
	Others (irrigation, animals, fertilizers)	**0.869904**	0.34072	0.871943	0.872824	0.127176191

Area	Support type	F1	Initial communality	Final communality	Specific variance	
Semi-arid	Monetary	**0.636556**	0.494949	0.405203	0.594797	
	Seeds	**0.754645**	0.710145	0.569489	0.430511	
	Machinery	**0.927924**	0.806316	0.861044	0.138956	
	Education	**0.869807**	0.804422	0.756564	0.243436	
	Others (irrigation, animals, fertilizers)	**0.863223**	0.776786	0.745154	0.254846	
Humid	Monetary	**0.671018**	0.474638	0.450265	0.549735	
	Seeds	**0.507419**	0.262673	0.257474	0.742526	
	Machinery	**0.988201**	0.981714	0.976541	0.023459	
	Education	**0.981327**	0.979104	0.963003	0.036997	
	Others (irrigation, animals, fertilizers)	**0.959075**	0.89	0.919825	0.080175	

F: load of a variable in a factor. Bold Figure: factors most responsible for the total degree of fluctuation of the considered variable.

In the arid region, the most important support type is machinery, followed by others, education, seeds and lastly monetary support. In the semi-arid regions, the most important support type for farmers is machinery, followed by education, others, seeds, and money. In the humid area, machinery loaded the highest followed by education, others, money and seeds.

The result from Table 8 further shows that the support needs vary between the agro-ecological zones. It, therefore, means that support has to be site-specific and relevant to the needs of the farmers in that area. Furthermore, it is worthwhile for each of the key players to work together based on their type of support and the area within which they are operating. For example, in the arid area where there is a strong correlation between education and seeds DAFF and NGOs can work closely together so that they pull their resources together and reach many more farmers.

Evidence from Table 9 shows there is a statistically significant difference between the various areas of the different agro-ecological zones. This is seen in the significant value of the chi-square 1 which is greater than the α-value 0.05. To reiterate this p-value for Wilks' G^2^ is compared with the α-value. With a p-value of 0.99 (Table 9) which is greater than α = 0.05, the null hypothesis which states that the means are independent is accepted.

**Table 9:** Test of significance.

Chi-square (Observed Value)	2.215
Chi-square (Critical value)	24.996
DF	15
p-value	1.000
alpha	0.05
Wilks' G^2^ (Observed value)	3.151
Wilks' G^2^ (Critical value)	24.996
DF	15
p-value	0.999
alpha	0.05

### 3.7 Discussions

The South African government has dedicated resources for enhancing farmer's resilience to climate change through various support schemes operating at national and provincial levels. Besides government interventions, the non-governmental sectors also play their role in assisting farmers. However, despite these best intentions, farmers such as those in Limpopo find it difficult to get access to these resources that could make a difference in their production and sustainability. What are therefore the implications for farmers not getting the support types that have been identified in Limpopo?

#### 3.7.1 Seed support

The availability of seeds to farmers can best be explained through the concept of seed security. This concept, as ingrained in the Seed Security Framework entails the need for a sufficient quantity of seed of target crops within reasonable proximity, and in time for sowing, periods to be made available for farmers (Sperling et al., 2006). Therefore, where farmers receive the needed seed support, they have the potential to improve yield. In the case of Limpopo, the most support received by farmers was in the form of seeds (72.5% of farmers in the arid, 57.5% in the semi-arid and 70% in the humid zones) as seen in Table 1. Looking at the support needs of the farmer, Based on the factor analysis, seeds are not among the most important support types for farmers, as they are ranked 4th in arid and semi-arid zones, whereas they are last in humid zones. This might mean that the farmers have means of which to get seeds and that seeds are not the most important input to booster their level of adaptation.

#### 3.7.2 Machinery support

Agricultural productivity is closely related to machinery and investments in all aspects of agricultural activity (Andriy and Prokopenko, 2017). In its *Least Developed Countries Report*, the United Nations indicated the level of mechanization in agricultural production as one of the major indicators of agricultural productivity (2015). According to Sims and Kienzle (2006) mechanization is a key input in any farming system. The aim of mechanization amongst others is to increase productivity per unit area through improved timeliness of farm operations; expansion of the areas under cultivation where land is available as well as the improvement of the quality of work and products. This might explain why in Table 9, machinery loaded the highest with regards to support type the farmers needed across all the three zones. Studies such as Agarwal (1981); Clare Bishop-Sambrook (2005) and Faleye et al. (2012) show that mechanization helps not only in increasing productivity but also in improving the quality of all the farm operations and final products. Furthermore, the FAO and World Bank (2009) reporting on agricultural production in the Kyrgyz Republic showed that lack of mechanization amongst other factors negatively affects aggregate agricultural production output.

Nevertheless, acquiring agricultural machinery alone cannot ensure productivity as well as climate change mitigation and adaptation without additional measures. This is seen in the study of Faleye et al. (2012) who posit that a number of minimum conditions of mechanization have to be in place in order to ensure efficient small farm functioning. These conditions include aspects of farm suitability, simple design and technology, affordability and accessibility and the provision of support services from the government. Unfortunately, amongst the smallholder farmers in Limpopo, most of these conditions are not met. Firstly as seen in Table 1, very few farmers had support in terms of machinery from the government. Secondly, the success of mechanization is dependent on the inclusion of agricultural extension and advisory efforts which are essential for the operationalization of any mechanization and sustainable farming system, mitigation and adaptive measures. However, this aspect is minimally meet given that in terms of educational support only 66.5% of farmers in the arid, 48.5% in the semi-arid and 37.5% in the humid zones are supported. This means only a small percentage of farmers are getting educational support and the majority would not have been able to function with machinery had they been given since they would have been lacking in knowledge on operating them. It is essential that investment is made in terms of agricultural machinery with an aggregate support system given that it has been proven to be effective and highly advantageous for agricultural and economic development in general (e.g. Stavytskyy and Prokopenko, 2017).

#### 3.7.3 Educational support

Nelson and Phelps (1966) posit that a better-educated farmer is quicker to adopt profitable new processes and products given that they are aware that the expected payoff from innovation is likely to be greater and the risk likely to be smaller. Education enhances a farmer's ability to know his alternatives, to know when and where to buy and sell, proper farm management procedures specific to the crops cultivated such as tillage, fertilizer application.

Most farmers in rural areas such as those in Limpopo are not privy to the most up-to-date information on efficient food production, cost-effective means of as well as the proper implementation of adaptation techniques to a changing climate. The question which arises with cases such as the resource-poor farmers in Limpopo is how they get the necessary education needed for optimum production and adaptive capacity to a changing climate. A possible solution that has also been cited by Rosegrant and Cline (2003) will be that of enhancing their understanding of new techniques and technologies and also providing them with any physical resources necessary for implementation. From that perspective, a look at the Strategic Plan for South African agriculture (National Department of Agriculture (NDA), 2001) shows that the national and provincial departments of agriculture are committed to the provision of extension support to land reform beneficiaries. Extension services are particularly necessary where plots are intensively farm because they cannot be expanded easily (Machethe and Mollel, 2000). In South Africa, agricultural extension services serve as an important link between small-scale farmers and the Department of Agriculture (Jacobs, 2003). This might explain why in Table 1, most support received by the farmers is from DAFF. Table 1 further shows that of the 600 farmers surveyed, only 58.33% of the farmers were receiving educational support. Across the zones, DAFF is supporting less than half of the population (46.5% in the arid, 28.5% in the semi-arid and 27.5% in the humid zones) and NGOs provide additional educational support (20% in the arid, 20% in the semi-arid and 10% in the humid zones). Furthermore, Table 6 shows there is a significant difference in the total number of farmers receiving educational support amongst the zones. Granting the South African government promotes access to agricultural extension and advisory services by the farmer, lack of access is still a reality at the grassroots level as seen in Limpopo. This situation can be as a result of vast numbers of people requiring assistance, the relatively few and inadequately trained and resourced extension workers (DAFF, 2012) thereby increasing officer to farmer ratios. DAFF estimates that the percentage of small-scale farmers reached through the extension service grew from 8% in 2010, but was still at just 14% in 2012/13 (DAFF, 2015). Also given that there is no regulatory framework within which the delivery of extension and advisory service takes place (DAFF, 2014) extension and advisory services face major challenges in the areas of relevance, efficiency, accountability and sustainability in South Africa (DAFF, 2014).

Given that extension is the conscious communication of information to help people form sound opinions and make good decisions (Van den Ban and Hawkins (1996); facilitates the access to knowledge, information, and technologies by farmers, farmers organizations and other market actors; facilitates farmer's interaction with research partners, educational institutions, agribusiness, and other relevant institutions; and further assists them in the development of their own technical, organizational and managerial skills and practices (Christoplos, 2010) then smallholder farmers in Limpopo are disadvantaged because they are not able to adequately benefit from educational support services through extension as has been seen by the low number of farmers receiving such support.

With regards to its importance to farmers in Limpopo, agricultural educational support services ranked third in the arid zone, second in the semi-arid and humid zones. Hence Extension programs aimed at increasing knowledge have the potential to increase the adoption of technology (Sasa, 2009; Oni et al., 2011). Furthermore, an increase in the frequency of extension visits to impart information could result in increased productivity and income generation (Ackello-Ogutu, 2011).

#### 3.7.4 Fertilizer, irrigation schemes

Fertilizers are commonly believed to be as important, and contribute up to 50% of the growth in output (Tomich et al., 1995). It is advocated that the fundamental ways in which agricultural productivity can be improved, especially in sub-Saharan Africa are to increase the productivity of agriculture through the use of farmer consultations and providing inputs, such as seeds, fertilizers, pesticides and the use of modern agricultural technology (Wanyama et al., 2009). Afzal and Ahmad (2009) show that stable fertilization leads to improved crop yields and agricultural income. However, these support factors are not adequately distributed amongst farmers in Limpopo. Of the farmers surveyed only 31.5% received these support types in the arid zone, 30% in the semi-arid and 34.5% in the humid zones respectively. In terms of support types needed, this support type loaded highly as seen in Table 9, loading as the second most important need in the arid, third most important in the semi-arid and humid zones respectively. The need for these type of support especially irrigation can be because of the climatic stresses especially drought which plagues this area as well as deficiencies in infrastructures and poor institutional support which (e.g Machethe et al., 2004; Tlou et al., 2006; Van Averbeke et al., 2011; Fanadzo, 2012) involved in irrigation projects across South Africa.

#### 3.7.5 Ease of accessing support institutions and credit facilities

There is no consensus on the extent to which monetary support as well as financial service provision such as credit, can help farmers adapt to a changing climate. This may be caused by the difficulty in measuring the impact of credit on poverty reduction. However, it is generally accepted that monetary support and financial services may assist farmers either directly or indirectly thereby having a spill down effect on the challenges faced by farmers in a changing climate. Zeller and Sharma (1998) believe that credit facilities may assist smallholder farmers to tap financial resources beyond their means thereby taking advantage of potentially profitable small business opportunities.

In Limpopo, the ease of getting support and accessing financial institutions is quite daunting to farmers. Table 2 shows that most farmers (33.3%) received two types of support. This is low considering that there are non-governmental and governmental institutions committed to supporting farmers in the area. From their responses as seen in Table 3, DAFF was the easiest institution to get support from followed by NGOs. Amongst the zone, the farmers experience similar levels of difficulties in getting support from the institution, showing that there is a problem various with role players carrying out their responsibilities adequately. Table 4 shows that cooperatives are the easiest to access in terms of obtaining loans. However, only 14%–16% of the farmers were able to access and obtain loans from cooperatives. Banks and microfinance institutions were not easily accessible. This situation might be as a result of farmers having to comply with certain requirements prior to receiving support. For example, the support criteria outlined in the Limpopo Department of Agriculture and Rural Development's (DARD) Farmer Support Policy provide a sense of the requirements when applying for and receiving grants (Limpopo Department of Agriculture and Rural Development (DARD), 2016). Small-scale farmers need to show proof of secure tenure, a completed farm assessment report and feasibility study, proof of business registration, operational records, a detailed business plan, proof of tax registration, and market contracts or letters of intent to buy. On the other hand Department of Rural Development and Land Reform's (DRDLR) One Household, One Hectare initiative, requires the farmer's production plan with cash flow projections before they are considered for financial support (DRDLR, 2016).

The inability to get adequate types and financial support places significant constraints for these farmers in both the opportunities forgone and their inability to mitigate risk and adapt to climate change. Furthermore, this constraint has proved to impede on the farmers' ability to innovate (Griffin et al., 2002, 2017) and hence can be concluded that they will not be open to suggestions of adaptation. According to Kandlinkar and Risbey (2000) since most farmers in Africa are operating under resource limitations: (a) Lack of credit, (b) Subsidies, and (c) Insurance will accelerate farmers' failure to meet transaction costs necessary to acquire adaptation measures as a result of unexpected weather patterns.

Studies such as that of Deressa et al. (2009) and Maddison (2007) have cited the difficulty of obtaining credit by farmers, as a crucial factor in determining the ability of farmers to adapt to climate change. In other settings, for example, credit markets are an important feature of Pakistan's rural agricultural economy owing to the range of different types of lenders that offer credit (Aleem, 1990). Therefore, easy access to credit may offset the effects of climate and play a crucial role in making the difference between being vulnerable and not. Improved access to agricultural credit and savings may help those with limited assets to invest in agricultural technology or land improvements, such as high-yielding seeds and chemical inputs that increase incomes. This opinion has been echoed by Asiedu and Fosu (2008) who think credit is a very important component in the modernization of agricultural activities. Furthermore, credit is seen as the backbone of any business, especially for agriculture, which has traditionally been a non-monetary activity for the rural population (Abedullah et al., 2009). Modern agricultural technology is necessary for national and economic development, and the use of such technology in rural economies is only possible when farmers are provided with credit for the purchase of modern technological inputs (Schultz, 1980). Hence agricultural credit is an integral part of the process of the modernization of agriculture.

Both commercial and subsistence farming is subject to climatic effects, but compared to commercial agriculture, smallholder farmers are more vulnerable to climate change and usually do not have access to financial instruments such as credit and insurance to hedge against climatic risk. As has been shown by studies such as Wiid and Ziervogel (2012) and Nhemachena and Hassan (2007), farmers with access to credit and markets have greater chances of adapting to changing climatic conditions. This is because being able to access affordable credit, farmer's financial resources are increased as well as their ability to meet transaction costs associated with the various adaptation options they might want to take. With more financial and other resources at their disposal farmers can change their management practices in response to changing climatic and other factors and will be able to make use of all the available information they might have on changing conditions both climatic and socioeconomic factors. For instance, with financial resources and access to markets, farmers can change their cropping calendar, chose appropriate cultivars, crop varieties, invest in new irrigation technologies, and change their practices to suit the forecasted and prevailing climatic conditions. At individual farm levels, adaptation will involve a combination of various individual responses and assumes that farmers have access to support infrastructures, alternative practices, and technologies available in the region. The support types available in Limpopo and the departments providing such supports appear good on paper but in implementing their stated objectives to a certain degree, they have failed.

## 4 Conclusion

The study revealed that both public and private institutions have significant roles to play in supporting farmers' adaptive capacity to a changing climate in the Limpopo Province. These roles include generation of appropriate educational, timely information and technology, ensuring that information and technology are available to the intended party for use, assistance with production inputs, facilitate access to financial institutions as well as supplying the adequate number of supports towards farmer's sustainability. Furthermore, with properly tailored policies and implementation of such policies, smallholder farmers can adjust to climate change and improve their crop production. To do this, policies need to factor in potential adaptations methods to climate change when farmers are given adequate support as well as taking into consideration what support types are relevant to a particular area. In this regard, interventions of the South African government should focus on the needs of farmers and not a one size fits all model for institutional support.

Hence, planning and financing adaptation to climate change requires an understanding of current conditions of what is available to farmers in the form of support in each area, how many support types are available to each farmer and how accessible these support institutions are. It also requires an understanding of how the various levels of governance enable or hinder local actors to improve their wellbeing. Knowing the what, how, when and where of climate change and the options for support towards adaptation, will allow for well-informed decision-making by farmers, policymakers and practitioners. These spatiotemporal dimensions and multi-actor perspectives promise relevant insights for achieving sustainability less haphazardly. Institutional frameworks and actions can thus hinder or facilitate adaptations (Dolan et al., 2001). In the case of Limpopo, institutions are falling short of their intended objectives of farmer support.

Based on the findings of this study the following recommendations can be put forward with regards to the various support factors:

• Given the current poor reach of extension services in Limpopo, better public-private partnerships should be developed and improve better coordination in such partnerships so that every farmer gets the necessary educational support.
• The government needs to implement a coherent extension policy to advance a varied system of extension providers. Given that climate change impacts are diverse, vulnerability is place-specific and adaptation requirements are diverse, there are various benefits from having various providers. This will mean the delivery of advice, technology, and innovations as well as facilitating services will be available from varied sources. Such a strategy requires putting in place of new and appropriate mechanisms for financing and/or co-financing the needs of farmers. Also, most importantly, it will require putting in place adequate mechanisms that will enhance the quality of services provided by diverse institutions. To effectively pursue such a strategy, the government needs to better understand the nature of the existing support services to design policies that will be supportive of a pluralistic system. It is also necessary that the government conducts a survey that will help in creating an inventory of key players and stakeholders, what they provide, whom they provide, assess the quality of the services they render before deciding on the type of reform necessary.
• Moreover, the success in fulling the public-private sector partnerships potential in South Africa is dependent on the creation of new ways to break down barriers to support systems through which the farmers can benefit such as less stringent rules for accessing support institutions or obtaining loans from financial institutions.

## Declarations

### Author contribution statement

P. N. Kephe: Conceived and designed the experiments; Performed the experiments; Analyzed and interpreted the data; Contributed reagents, materials, analysis tools or data; Wrote the paper.

K. K. Ayisi, B. M. Petja: Conceived and designed the experiments; Contributed reagents, materials, analysis tools or data; Wrote the paper.

### Funding statement

This work was supported by the University of Limpopo (UL) and the VLIR-UOS collaboration.

### Competing interest statement

The authors declare no conflict of interest.

### Additional information

No additional information is available for this paper.

## Appendix A Supplementary data

Questionnaire Edited _spl_2_spl_ Questionnaire Edited _spl_2_spl_
