# Supplementary material for: A decision support system for institutional support to farmers in the face of climate change challenges in Limpopo province
Source: Heliyon. 2020 Nov 3;6(11):e04989. doi: 10.1016/j.heliyon.2020.e04989 (PMC7644883; doi:10.1016/j.heliyon.2020.e04989)
Supplement: Questionnaire Edited _spl_2_spl_ [file mmc1.docx]

As part of the study: **RISK AND VULNERABILITY ANALYSIS OF DRYLAND AGRICULTURE UNDER PROJECTED CLIMATE CHANGE: ADAPTIVE RESPONSE IN SOUTH AFRICAN SUMMER RAINFALL AREAS we**  would like to invite you to complete the attached survey to inform our understanding of the vulnerability of farmers in Limpopo to climate change and variability. By participating, you will have the opportunity to provide important information about your experience as a farmer faced with the challenges of climate change and variability and will help us to give suggestions on adaptation measures. All the information you provide will be kept completely confidential. No reference will be made in written or oral materials that could link you to the study. In reports, the information you give us will be combined with what we get from everyone who participates in these interviews. Your participation in the interviews is completely voluntary.

Thank you for your participation!

**SECTION A: BACKGROUND INFORMATION**

**A1. Age…………………………………………………………**

**A2. Sex.........................................................................................**

**A3. Locality……………………………………………………..**

**A4. Name of farm………………………………………………**

**A5. Size of Farm……………………………………………….**

**SECTION B: AGRONOMIC PRACTICES AND CROP PRODUCTION**

**B1. How long have you been farming?....................................................( in years)**

**B2. Which of the following crops do you grow?**

| Groundnuts | 1 |  |
| --- | --- | --- |
| Soybeans | 2 |  |
| Sunflower | 3 |  |
| Others | 4 |  |

**B3. What tillage system do you use**?

| No-tillage | 1 |  |
| --- | --- | --- |
| Mulch tillage: | 2 |  |
| Strip or zonal tillage: | 3 |  |
| Ridge till: | 4 |  |
| Reduced or minimum tillage: | 5 |  |
| Hand digging of entire field | 6 |  |
| Planting basins | 7 |  |
| If other specify | 8 |  |

**B4. What varieties of the following crops do you grow?**

| **B4.1** Groundnuts | 1 | Akwa (254) | | 2 | Anel (254) | 3 | Billy (254) | 4 | Mwenje (1137) | 21 | Unknown |
| --- | --- | --- | --- | --- | --- | --- | --- | --- | --- | --- | --- |
|  | 5 | Nyanda (1173) | | 6 | Phb 95Y40 R (411 | 7 | Inkanyezi (959) | 8 | Phb 95Y41 R (411) |  |  |
|  | 9 | Phb 96B01 R (411 | | 10 | PAN 9212 | 11 | Phb 95B53 R (411) | 12 | Kangwane Red(254) |  |  |
|  | 13 | Harts (254 | | 14 | Phb 95Y20 R (411) | 15 | JL 24 (959 | 16 | Rambo (254) |  |  |
|  | 17 | Kwarts (254) | | 18 | Sellie……Tufa (254) | 19 | SA Juweel (254) | 20 | OtherSpecify |  |  |
| **B4.2** Soybean: | 1 | SNK 500 (24) | 2 | | Dundee (254) | 3 | Jimmy (254) | 4 | Kiaat (489) | 21 | Unknown |
|  | 5 | Egret (254) | 6 | | Mukwa (489 | 7 | Sonop (150) | 8 | Stork (254 |  |  |
|  | 9 | Maruti (305) | 10 | | Dumela (305) | 11 | Mopanie (489) | 12 | Knap (150) |  |  |
|  | 13 | Tambotie (489 | 14 | | LS 678 (484)…… | 15 | LS 677 (484) | 16 | PAN 626 (1412) |  |  |
|  | 17 | PAN 809 (1412) | 18 | | PAN 660 (1412) | 19 | PAN 1669 (1412 | 20 | Other Specify |  |  |
| **B4.3** Sunflower | 1 |  | 2 | | HV 3037 (254) | 3 | Sirena (1421) | 4 | AFG 271 (1) | 21 | Unknown |
|  | 5 | Hysun 3.33 (1421) | 6 | | AGSUN 4672 (254) | 7 | AFG 272 (1) | 8 | Hysun 346 (1421) |  |  |
|  | 9 | AGSUN 4683 (254) | 10 | | AGSUN 5261 (1) | 11 | Hysun 3.34 (1421) | 12 | PAN 7034 (1412 |  |  |
|  | 13 | AGSUN 5282 (1) | 14 | | PAN 7001 (1412) | 15 | DK 4040 (80) | 16 | NK FERTI (809 |  |  |
|  | 17 | ADAGIO CL (809 | 18 | | PAN 7031 (1412) | 19 | DKF 68-22 (80 | 20 | Other Specify |  |  |

**B5. During planting, are the row spacing random or non-random, if non-random what spacing?**

| **B5.1** Row spacing for Groundnuts | **(1)** Random |  | **B5.1.1** Spacing | (1) Length (cm) | **(2)** Breadth (cm) |
| --- | --- | --- | --- | --- | --- |
| **B5.2** row spacing for soybeans | 1. **Random** |  | **b.5.2.1** Spacing | (1) Length (cm) | **(2)** Breadth (cm) |
| B5.3. Sunflower | 1. **Random** |  | **B5.3.1** Spacing | (1) Length (cm) | **(2)** Breadth (cm) |

**B6.** **When do/did you plant?**

| **B6.1** Planting date for groundnut | **[1]** September |  | **[2]**October |  | **[3]**November | **[4]** December |
| --- | --- | --- | --- | --- | --- | --- |
|  |  |  |  |  |  |  |
| **B6.2** Planting date for soya beans | **[1]** September |  | **[2]**October |  | **[3]**November | **[4]** December |
|  |  |  |  |  |  |  |
| **B6.3** Planting date for sunflower | **[1]** September |  | **[2]**October |  | **[3]**November | **[4]** December |
|  |  |  |  |  |  |  |

**B7. When do you apply fertilizer?**

| Fertiliser application | **[1]** Before planting | **[2]** Days after planting | **[4]** Flowering | **[5] Do not apply** |
| --- | --- | --- | --- | --- |
| **B7.1** Groundnut |  |  |  |  |
| **B7.2** Soya bean |  |  |  |  |
| **B7.3** Sunflower |  |  |  |  |

**B8. What types of fertilizer do you use, if any?**

| **B8.1** Groundnut | **[1]** Nitrogen (N) |  | **[2]** Phosphate (P_2_O_5_) |  | **[3]** Potash (K_2_O) | **[4]** Ammonium Nitrate or Urea | **[5]**Compound D | **none** |
| --- | --- | --- | --- | --- | --- | --- | --- | --- |
|  |  |  |  |  |  |  |  |  |
| **B8.2** Soya beans | **[1]** Nitrogen (N) |  | **[2]** Phosphate (P_2_O_5_) |  | **[3]** Potash (K_2_O) | **[4]** Ammonium Nitrate or Urea |  |  |
|  |  |  |  |  |  |  |  |  |
| **B8.3 S**unflower | **[1]** |  | **[2]** Phosphate (P_2_O_5_) |  | **[3]** Potash (K_2_O) | **[4]** Ammonium Nitrate or Urea |  |  |
|  |  |  |  |  |  |  |  |  |

**9. What is the rate of fertilizer application?**

| **B9.1** Groundnut | **[1]** Nitrogen (N) |  | **[2]** Phosphate (P_2_O_5_) |  | **[3]** Potash (K_2_O) | **[4]** Ammonium Nitrate or Urea | **[5]**Compound D |
| --- | --- | --- | --- | --- | --- | --- | --- |
|  |  |  |  |  |  |  |  |
| **B9.2** Soya beans | **[1]** Nitrogen (N) |  | **[2]** Phosphate (P_2_O_5_) |  | **[3]** Potash (K_2_O) | **[4]** Ammonium Nitrate or Urea |  |
|  |  |  |  |  |  |  |  |
| **B9.3 S**unflower | **[1]** |  | **[2]** Phosphate (P_2_O_5_) |  | **[3]** Potash (K_2_O) | **[4]** Ammonium Nitrate or Urea |  |
|  |  |  |  |  |  |  |  |

**B10. What other means of fertilization do you employ?**

| **B10.1** Groundnut | **[1]** Kraal manure |  | **[2]** compost | **[3]** Leaf litter | **[** **4]**Ash | **[ 5]**  **Crop** | **[ 6]**  **others** |
| --- | --- | --- | --- | --- | --- | --- | --- |
|  |  |  |  |  |  |  |  |
| **B10.2** Soya beans | **[1]** |  | **[2]** | **[3]** |  |  |  |
|  |  |  |  |  |  |  |  |
| **B10.3 S**unflower | **[1]** |  | **[2]** | **[3]** |  |  |  |
|  |  |  |  |  |  |  |  |

**11. What is the rate of fertilizer application?**

| **B11.1** Groundnut | **[1]** Kraal manure |  | **[2]** compost |  | **[3]** Leaf litter | **[4]**Ash | **[ 5]**  **Unknown** |
| --- | --- | --- | --- | --- | --- | --- | --- |
|  |  |  |  |  |  |  |  |
| **B11.2** Soya beans | **[1]** |  | **[2]** |  | **[3]** |  |  |
|  |  |  |  |  |  |  |  |
| **B11.3 S**unflower | **[1]** |  | **[2]** |  | **[3]** |  |  |
|  |  |  |  |  |  |  |  |

| **B12**. **Do you apply herbicides, pesticides, fungicide?**   \|  \| **Yes[1]** \| **NO [2]** \| \| --- \| --- \| --- \| \| **12.1 Herbicide** \|  \|  \| \| **12.2 Pesticide** \|  \|  \| \| **12.3 fungicide** \|  \|  \| |  |  |  |  |
| --- | --- | --- | --- | --- | --- | --- | --- | --- | --- | --- | --- | --- | --- | --- | --- | --- |

**B. 13 Do you control weeds on your farm? Yes [1]🞏 No [2]🞏**

B. 13**.1. How effective is your weed control**

| Degree of effectiveness | [1] very effective | [2] somewhat effective | [3] not effective at all |
| --- | --- | --- | --- |
|  |  |  |  |

**B13. 2 How often do you weed the field from planting to harvesting?**

| crop | **[ 1]** Once | **[2]** twice | **[3] Thrice** |
| --- | --- | --- | --- |
| **B13.2..1** Groundnut |  |  |  |
| **B13.2..2** Soya bean |  |  |  |
| **B13.2.3** Sunflower |  |  |  |

**B14. What methods of weeding do you use?**

| Pesticide application |  | **[ 1]** chemical | | **[2]** manual Hoe | **[3]** mechanical (tractor) |
| --- | --- | --- | --- | --- | --- |
| **B14.1** Groundnut |  | |  |  |  |
| **B14.2** Soya bean |  | |  |  |  |
| **B14.3** Sunflower |  | |  |  |  |

**B15. Do you use any water management techniques? Yes [1]🞏 No [2]🞏**

**B. 15.1 If Yes, which water management techniques do you use?**

|  | **[1]** cover crops | **[2]** Contour ploughing | **[3]** Ridging | **[4]** Deep weeding | **[5]** Pot holding | **A[6]** mulching | **[7]** furrow Drainage | **[8] if** other Specify |
| --- | --- | --- | --- | --- | --- | --- | --- | --- |
| **B15.1.1** Groundnut |  |  |  |  |  |  |  |  |
| **B15.1.2** Soya bean |  |  |  |  |  |  |  |  |
| **B15.1.3** Sunflower |  |  |  |  |  |  |  |  |

**B16. Which crop production factors influence your investment decisions?**

| **crops** | **Factors** | **Constrain(A)** | **Non constrain(B)** |
| --- | --- | --- | --- |
| **B16.1** Groundnut | 1. **Input availability** |  |  |
|  | 1. **Labour** |  |  |
|  | 1. **Food security** |  |  |
|  | 1. **Draft power** |  |  |
|  | 1. **rainfall** |  |  |
|  | 1. **floods** |  |  |
|  | 1. **[Temperature** |  |  |
|  | 1. **water(irrigation)** |  |  |
|  | 1. **irrigation equipment** |  |  |
|  | 1. **Implements** |  |  |
|  | 1. **Cash** |  |  |
| **B16.2** Soya bean | 1. **Input availability** |  |  |
|  | 1. **Labour** |  |  |
|  | 1. **Food security** |  |  |
|  | 1. **Draft power** |  |  |
|  | 1. **Rainfall** |  |  |
|  | 1. **Flood** |  |  |
|  | 1. **Temperature** |  |  |
|  | 1. **Water(irrigation)** |  |  |
|  | 1. **Irrigation equipment** |  |  |
|  | 1. **Implements** |  |  |
|  | 1. **Cash** |  |  |
| **B16.3** Sunflower | 1. **Input availability** |  |  |
|  | 1. **Labour** |  |  |
|  | 1. **Food security** |  |  |
|  | 1. **Draft power** |  |  |
|  | 1. **Rainfall** |  |  |
|  | 1. **flood** |  |  |
|  | 1. **Temperature** |  |  |
|  | 1. **Water (irrigation)** |  |  |
|  | 1. **Irrigation equipment** |  |  |
|  | 1. **Implements** |  |  |
|  | 1. **Cash** |  |  |

**B17. Which cropping decisions are influenced by climate?**

|  | **[1]** Planting date | **[2]** Fertilizer application | **[3]** Choice of crop | **[4]** Deep weeding | **[5]**Variety to grow | **[6]** water | **[7] others** |
| --- | --- | --- | --- | --- | --- | --- | --- |
| **B17.1** Groundnut |  |  |  |  |  |  |  |
| **B17.2** Soya bean |  |  |  |  |  |  |  |
| **B17.3** Sunflower |  |  |  |  |  |  |  |

**B18. Are there any deviations from usual agronomic practices, this year?** **Yes [1]🞏 No [2]🞏**

**B18.1 if yes, what were the deviations?**

|  | **[1]** Increased range of crops | **[2]** Reduced range of crops | **[3]** More area planted | **[4]** Less area planted | **[5]** Different varieties | **[6]** Conservation tillage | **[7]** Fertilizer applied at planting | **[8] if** other Specify |
| --- | --- | --- | --- | --- | --- | --- | --- | --- |
| **B18.1.1**Groundnut |  |  |  |  |  |  |  |  |
| **B18.1.2** Soya bean |  |  |  |  |  |  |  |  |
| **B18.1.3** Sunflower |  |  |  |  |  |  |  |  |
| **B 18.1.4** |  |  |  |  |  |  |  |  |

**B19. What are the reasons for deviations in B16 above apart from climatic factors?**

|  | **[1]** Seed availability | **[2]** Fertilizer | **[3]** water | **[4] temperature** | **[5] if others specify** |
| --- | --- | --- | --- | --- | --- |
| **B19.1** Groundnut |  |  |  |  |  |
| **B19.2** Soya bean |  |  |  |  |  |
| **B19.3** Sunflower |  |  |  |  |  |

**SECTION C: CONSTRAINTS ON AGRONOMIC PRACTICES AND CROP PRODUCTION CAUSED BY CLIMATE CHANGE AND VARIABILITY**

**C1**. Have you noticed any changes in the general weather from the time you started farming? Yes [1]🞏 No [2]🞏

C**2**. If yes, how?

| Short season length | 1 |  |
| --- | --- | --- |
| Low rainfall | 2 |  |
| Mid-season dry spells | 3 |  |
| Abrupt end of season | 4 |  |
| Late rains | 5 |  |
| High rainfall (Higher than normal) | 6 |  |

**C3. Are these changes in the weather apparent from year to year?** Yes [1]🞏 No [2]🞏

**C4.If “yes”, how has it affected you in the past farming season 2016/2017?**

|  | **[1]** Increased range of crops | **[2]** Reduced range of crops | **[3]** More area planted | **[4]** Less area planted | **[5]**Different varieties | **[6]** Conservation tillage | **[7]** Fertilizer not applied at planting | **[8] if** other Specify |
| --- | --- | --- | --- | --- | --- | --- | --- | --- |
| **C4.1** Groundnut |  |  |  |  |  |  |  |  |
| **C4.2** Soya bean |  |  |  |  |  |  |  |  |
| **C4.3** Sunflower |  |  |  |  |  |  |  |  |

**C5. Do these changes in the weather impact your farming activities? Yes [1]🞏 No [2]🞏**

**C5.1. If yes, how so?**

|  | **[1]** Planting date | **[2]** Fertilizer application | **[3]** Choice of crop | **[4]** Varieties to grow | **[5]** If other specify |
| --- | --- | --- | --- | --- | --- |
| **C5.1.1** Groundnut |  |  |  |  |  |
| **C5.1.2** Soya bean |  |  |  |  |  |
| **C5.1.3** Sunflower |  |  |  |  |  |

**C6.Have these changes in activities change drastically since you started farming?** Yes [1]🞏 No [2]🞏

**C7. Changes in activities from year to year?** Yes [1] 🞏 No [2]🞏

**C8. Which climatic thresholds have affected you the most?**

| Floods | 1 |  |
| --- | --- | --- |
| Droughts | 2 |  |
| Hail | 3 |  |
| Water logging | 4 |  |
| Snow | 5 |  |
| Others (Name) | 6 |  |

**SECTION D: COPING /ADAPTATION STRATEGIES TO CLIMATE VARIABILITY/ CHANGE**

**D1.** **Have you any ways to deal with the extreme event mentioned in C8 above?** Yes [1]🞏 No [2]🞏

**D2. If yes,** Have your method(s) of dealing with the above mentioned event involve changes in practices/strategies on the farm since you started farming? Yes [1]🞏 No [2]🞏

**D3. If “yes”, how and what are the methods _______________________________________________________________________________________________________________________________________________________________________________________________________________________________D4.** Did you notice any changes due to the response method employed in (D3)? Yes [1]🞏 No [2]🞏

**D5. If yes what were these changes?**

**D6.**How do you manage changes in:

Short season length: _________________________________________________________________________________________________________________________________________________________________________________________________________________

Low rainfall: _______________________________________________________________________________________________________________________________________________________________________________________________________________

Mid-season dry spells: _________________________________________________________________________________________________________________________________________________________________________________________________________________

Abrupt end of season: _________________________________________________________________________________________________________________________________________________________________________________________________________________

Late rains: _________________________________________________________________________________________________________________________________________________________________________________________________________________

High rainfall (Higher than normal): ________________________________________________________________________________________________________________________________________________________________________________________________________

Water-logging: _________________________________________________________________________________________________________________________________________________________________________________________________________________

**D7.What other sources of income do you have?**

| Other commercial activities | 1 |  |
| --- | --- | --- |
| Employment | 2 |  |
| Animal | 3 |  |
| Pension | 4 |  |
| Child grant | 5 |  |

**D8. What is the size of your household?**

| **D8.1. Gender** | | **M[1]** | | | **F[2]** | | | |
| --- | --- | --- | --- | --- | --- | --- | --- | --- |
| **Total** | |  | | |  | | | |
| **D8.2. Age (Years)** | **0-15 [1]** | | **16-26 [2]** | **27-37 [3]** | | **38 -48 [4]** | **49-59 [5]** | **Above 60 [6]** |
|  |  | |  |  | |  |  |  |

| **D.8.3. Marital Status** | **Single [1]** | **Married [2]** | **Divorced [3]** | **Widowed.[4]** |
| --- | --- | --- | --- | --- |
|  |  |  |  |  |

**D9. Are there any other members of your extended family dependent on you?** **Yes [1]🞏 No [2]🞏**

**D9.1 if yes how many__________**

**D10. What is the predominant livelihood of your community?** **___________________________________________________________________________________________________________**

**D11. What infrastructure and institutional arrangements are in place to support farmers?**

| institution | Monetary[1] | Seeds[2] | Machinery[3] | Educational support[4] | Others (Nirrigation,fertilizers, animals) [5] |
| --- | --- | --- | --- | --- | --- |
| 11.1 Agro finance |  |  |  |  |  |
| 11.2 Banks |  |  |  |  |  |
| 11.3 DAFF |  |  |  |  |  |
| 11.4 others |  |  |  |  |  |

**D12. Are these institutions easily accessible?** **Yes [1]🞏 No [2]🞏**

**D13. How easy is it for you to get loans from financial institutions?**

|  | **[1]**  **Very easy** | **[2]**  **Somewhat easy** | **[3]**  **easy** | **[4]**  **Not very easy** | **[5]**  **Not easy at all** |
| --- | --- | --- | --- | --- | --- |
| **Agro finance** |  |  |  |  |  |
| **Banks** |  |  |  |  |  |
| **Cooperatives** |  |  |  |  |  |

**D 14. According to you what are the most important changes best situated to maintain production of your crops in the face of climate change: Rank them in order of importance with 1 being the most important**

| **Practices categories** | **Description** | **code** | **Rank(1-5)** |
| --- | --- | --- | --- |
| **D14.1 On-farm Management** | Apply fertilizers according to fertilizer recommendations | [1] |  |
|  | Apply fertilizer that breaks down and releases nutrients slowly | [2] |  |
|  | Changing crop produced to another | [3] |  |
|  | Feed crop residues to livestock | [4] |  |
|  | Changing plant density | [5] |  |
| **D 14.2**  **New technologies** | Adopt drought tolerant and fast maturing cultivars | [1] |  |
|  | Changing in tools used for faring | [2] |  |
| **D14.3 Conservation agriculture** | Adopt no-till production | [1] |  |
|  | Adopt Ripper tillage production | [2] |  |
|  | Apply crop residue as a mulch to bare soil | [3] |  |
| **D14.4**  **Diversification on and beyond the Farm** | Intercrop with legumes | [1] |  |
|  | Intercrop crop with trees | [2] |  |
|  | Changing from crop production to livestock and dairy production | [3] |  |
|  | Shift from farming to non-farming activities | [4] |  |
| **Different dating of farm practices** | Changing planting date | [1] |  |

**SECTION E. REVENUE**

**E.1 Due to changes experienced in section B and C above, have you experienced any changes to**

| **crop** | **factor** | **Yes[1]** | **No[2]** | **Increase[1]** | **decrease[2]** |
| --- | --- | --- | --- | --- | --- |
| **E 1.1 Groundnut** | **Yield output** |  |  |  |  |
|  | **Cost of production** |  |  |  |  |
|  | **Revenue** |  |  |  |  |
| **E1.2 Soy bean** | **Yield output** |  |  |  |  |
|  | **Cost of production** |  |  |  |  |
|  | **Revenue** |  |  |  |  |
| **E1.3 Sunflower** | **Yield output** |  |  |  |  |
|  | **Cost of production** |  |  |  |  |
|  | **Revenue [** |  |  |  |  |
| **E1.4 others** | **Yield output** |  |  |  |  |
|  | **Cost of production** |  |  |  |  |
|  | **Revenue [** |  |  |  |  |

**E2 What are the measured changes of these factors in the past three cropping season**

| **crop** | **factor** | **2014/2015** | **2015/2016** | **2016/2017** |
| --- | --- | --- | --- | --- |
| **E2.1 Groundnut** | **Yield output (t/ha)** |  |  |  |
|  | **Cost of production(R)** |  |  |  |
|  | **Revenue (R)** |  |  |  |
| **E2.2 Soy bean** | **Yield output (t/ha)** |  |  |  |
|  | **Cost of production (R)** |  |  |  |
|  | **Revenue ( R)** |  |  |  |
| **E2.3 Sunflower** | **Yield output (T/Ha)** |  |  |  |
|  | **Cost of production (R )** |  |  |  |
|  | **Revenue (R)** |  |  |  |
| **others** |  |  |  |  |

| **Item** |  | **2015** | **2016** | **2017** |
| --- | --- | --- | --- | --- |
| **Planting Date** | **Groundnut** |  |  |  |
|  | **Soybean** |  |  |  |
|  | **Sunflower** |  |  |  |
| **Flowering Date** | **Groundnut** |  |  |  |
|  | **Soybean** |  |  |  |
|  | **Sunflower** |  |  |  |
| **Harvesting date** | **Groundnut** |  |  |  |
|  | **Soybean** |  |  |  |
|  | **Sunflower** |  |  |  |
